# Plant Stem Bark Extractivism in the Northeast Semiarid Region of Brazil: A New Aport to Utilitarian Redundancy Model

**DOI:** 10.1155/2012/543207

**Published:** 2012-01-26

**Authors:** Washington Soares Ferreira Júnior, Clarissa Fernanda Queiroz Siqueira, Ulysses Paulino de Albuquerque

**Affiliations:** ^1^Laboratory of Applied Ethnobotany, Department of Biology, Federal Rural University of Pernambuco, 52171-900 Recife, PE, Brazil; ^2^Natural Products Laboratory, Department of Pharmaceutical Sciences, Federal University of Pernambuco, 50740-521 Recife, PE, Brazil

## Abstract

We use the model of utilitarian redundancy as a basis for research. This model provides predictions that have not been tested by other research. In this sense, we sought to investigate the stem bark extraction between preferred and less-preferred species by a rural community in Caatinga environment. In addition, we sought to explain local preferences to observe if preferred plants have a higher content of tannins than less-preferred species. For this, we selected seven preferred species and seven less-preferred species from information obtained from semistructured interviews applied to 49 informants. Three areas of vegetation around the community were also selected, in which individuals were tagged, and were measured the diameter at ground level (DGL) diameter at breast height (DBH), and measurements of available and extracted bark areas. Samples of bark of the species were also collected for the evaluation of tannin content, obtained by the method of radial diffusion. From the results, the preferred species showed a greater area of bark removed. However, the tannin content showed no significant differences between preferred and less-preferred plants. These results show there is a relationship between preference and use, but this preference is not related to the total tannins content.

## 1. Introduction

Several ethnobotanical studies have observed a great familiarity in the use of plants by local populations [1–3], indicating that this knowledge can provide information for the management and conservation of plant species. In the Brazilian semiarid region, a broad knowledge and use of medicinal plants by local people in the Caatinga ecosystem has been documented [4–6], including studies investigating the use of medicinal plants and its implications for conservation [7, 8].

One application of ethnobotany to the conservation and management of useful species of the Caatinga is the utilitarian redundancy model proposed by de Albuquerque and Oliveira [9]. Aiming to investigate the use pressure from local populations on plant species, the model presents two situations. The first indicates that in a redundant utilitarian category, that is, with a large number of species that serve the same function or use category, there is a decrease in the use pressure on a given species, as this pressure is spread out among a greater number of species. A second situation occurs in a redundant category: in the presence of preferred species, use pressure is shifted to these species. However, this model was proposed for a theoretical situation and lacks specific tests of its predictions.

The present study seeks to test this model's predictions by investigating whether there is indeed a shift of use pressure toward preferred species. We investigated the preferences of local communities for specific native plants for the treatment of inflammations because evidence shows that plants native to the Caatinga are preferred for medicinal use and that inflammation is one of the most important ailments for many local communities [6]. For example, several ethnobotanical studies have indicated native Caatinga species as priorities for future conservation programs considering the high use pressure that may be associated [7, 9, 10]. In addition, several studies have reported that many species of the Caatinga are used as anti-inflammatory, given the importance of this use category for various local communities [4, 6]. In this case, it is interesting to study the relationship between preference and use pressure on species used as anti-inflammatory, which provides theoretical implications for a better understanding of the utilitarian redundancy model, and also conservation implications for future management programs. Accordingly, we tested the hypothesis that preferred plants as anti-inflammatory by a local population of Caatinga suffer a greater use pressure than less-preferred plants.

Additionally, this study seeks to understand these preferences by analyzing the chemical compounds present in the bark of the species. There are several compounds tested in the literature which have an anti-inflammatory activity [11]. However, other researchers have observed that Caatinga species mentioned as anti-inflammatory by local populations have tannins in the bark [6, 12], indicating that this is an important class of compounds in the medicinal use of Caatinga species [7, 13–15]. Based on these observations, this research also seeks to test the hypothesis that tannin content represents a selection factor in the choice of species preferred by local people, while preferred plants will present a higher tannin content than less-preferred plants.

## 2. Materials and Methods

### 2.1. Study Area

This research was conducted in the municipality of Altinho in a semiarid region of Pernambuco (northeastern Brazil), located 163.8 km from the state capital. The population of the municipality of Altinho is approximately 22,363 people, with a territory of 454 km^2^ [16]; the urban population is made up of approximately 13,000 people and the rural population approximately 9,500 people [16]. The municipality is situated in a Caatinga environment, with vegetation characteristic of semiarid northeastern Brazil, including deciduous and semideciduous species [15].

The studied community, known as *Carão*, is located 16 km from the center of the municipality of Altinho and consists of 189 people living on 61 houses [17]. The center of the community resembles a village, with houses very close to each other, although most of the houses outside the center are very dispersed. The streets are unpaved, which hinders the access to the community, and the houses are made of brick [17]. Recently, a system was built to carry water from local rivers to homes, but many residents still use cisterns to capture rainwater or carry water from local rivers to their homes using barrels [17].

There are vegetated areas surrounding the community that provide timber and nontimber resources to residents. The community is located next to a mountain, known as “Serra do Letreiro”, which includes some areas of vegetation that, according to residents, have suffered no human impact because of their inaccessibility or inadequacy for cultivation, although much of the mountain's vegetation has been disturbed. The areas at the top of the “Serra do Letreiro” are known as “Chã da Serra” and are used as corn- and bean-growing areas, the main income-generating activities of the community, as well as pasture areas [18]. Other important resource areas are the mountain base, known as “base of slope”, and “pastures” and “plantations” areas located close to the residences [18].

### 2.2. Ethnobotanical Data

This research started from a database built by previous ethnobotanical studies conducted in the study area [12, 14, 15, 17, 19–21]. From this database, we selected the informants who participated in this study, provided that they cited at least one plant useful for the treatment of inflammation, resulting in the selection of 49 informants more than 18 years old and the use of 24 native plants indicated for the treatment of inflammation by at least one informant. Species collected by authors from previous studies were deposited at the Herbarium Professor Vasconcelos Sobrinho (PEUFR) of the Universidade Federal Rural de Pernambuco (UFRPE).

The first stage of this research was based on data from a previous survey conducted in the area [22], which was based in semistructured interviews conducted with the selected informants. In the interviews, the checklist-interview technique was used through the presentation of visual stimuli to the informants, using photographs of studied plants [23]. Informants were asked to rank the photographs (plants) according to their preference [24] for each type of inflammatory condition mentioned during the interview. Plants presented in the top positions in various rankings were considered to be preferred, while those listed as lesspreferred took the bottom positions in various rankings. For this, a salience analysis was used to observe the plants positions in the rankings and define the preferred and less-preferred plants. Preference is here defined as the conscious choice of informants of a particular plant species over others that are equally available [25]. 

**Figure 1:**
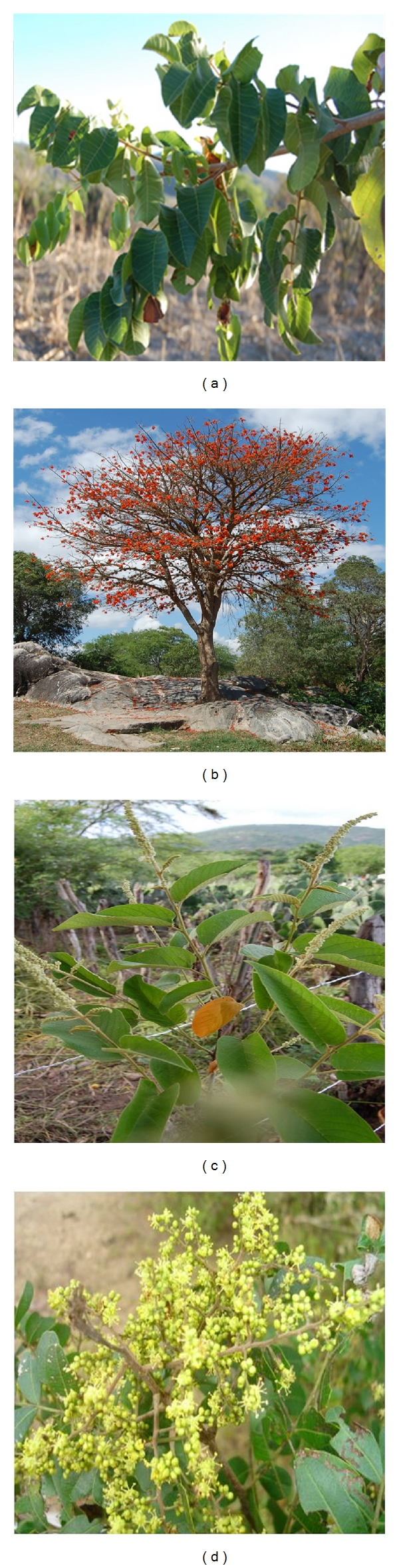
Photos of some plants selected for this study, in the rural community of Carão, Altinho, northeastern Brazil. (a)* Myracrodruon urundeuva* Allemão “aroeira”; (b)* Erythrina velutina* Willd. “mulungu”; (c)* Croton blanchetianus* Baill. “marmeleiro”; (d)* Schinopsis brasiliensis* Engl. “baraúna”. Photos: F. J. Vieira.

For the next steps, related to the extraction of stem bark and to measuring tannin content, we selected for study only those plants, both preferred and less-preferred, for which the bark was the primary source of reported anti-inflammatory properties. This selection was necessary because the hypothesis being tested depended on the use of stem bark as anti-inflammatory. In this case, measures of extraction of stem bark were made in species in which this plant part would be extracted mainly to treat inflammation, since the stem bark of several species in the community is used for various purposes. To select these plants, the database was checked for the number of cited uses for the bark of preferred and less-preferred species. Accordingly, 14 plants were selected (Table 1) that showed more than 50% of the number of uses cited indicating the use of its stem bark in inflammatory diseases.  Figure 1 shows some of the studied species.

### 2.3. Evidence of Stem Bark Extraction from Preferred and Less-Preferred Plants

For this component of the study, vegetation areas close to the community were selected to test the first hypothesis. There were tours with two local experts, nominated by the community as knowledgeable about the vegetation areas' use history. Three areas were selected, from which, according to local experts, several people from the community use to remove the bark of local plants to treat diseases.

**Figure 2:**
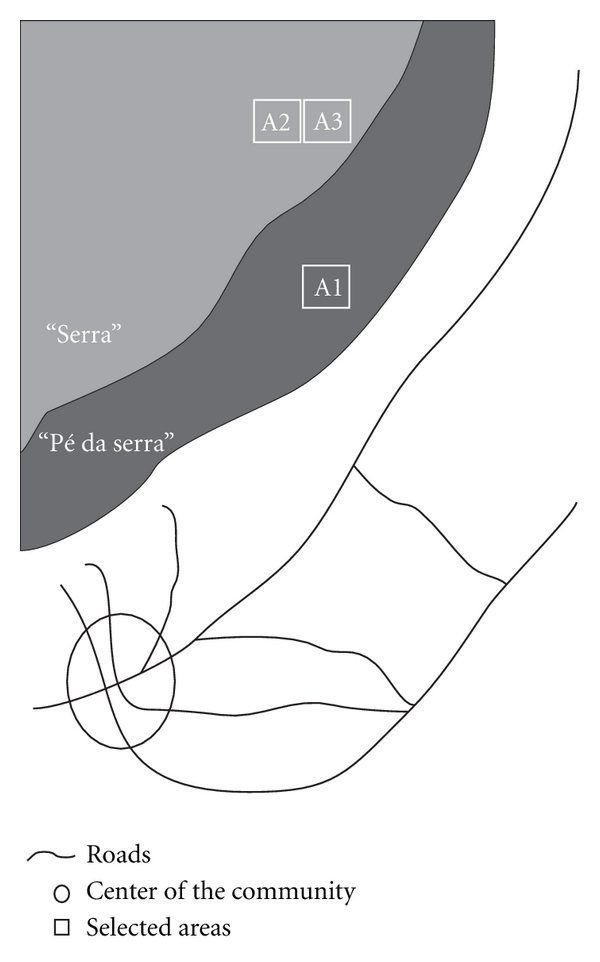
Representation of the study areas in the community of Carão, Altinho, northeastern Brazil. Area 1 (A1) is located in “Pé da Serra” and about 950 m away from the center of the community. Areas 2 and 3 (A2 and A3) are located in the region of “Serra”, about 1.4 km from the center.

The first selected area (Area I)—S 8° 34′ 80′′, W 36° 05′ 28′′—has approximately 13,819 m^2^ and is located in the “Pé da Serra”, very close to the community, approximately 950 m from the center. The second selected area (Area II) —S 8° 34′ 50′′, W 36° 05′ 35′′—has approximately 2,195 m^2^ and is located in the “Serra” region, approximately 1.4 km from the center of the community and about 550 m from Area I. The second area is located along the route that many locals take to their properties in the “Serra”. The third selected area (Area III) is only 103 m from the center of Area II and is also located in the region of “Serra”. Area III has an area of approximately 2,092 m², at S 8° 34′ 52′′, W 36° 05′ 29′′ (see Figure 2).

**Figure 3:**
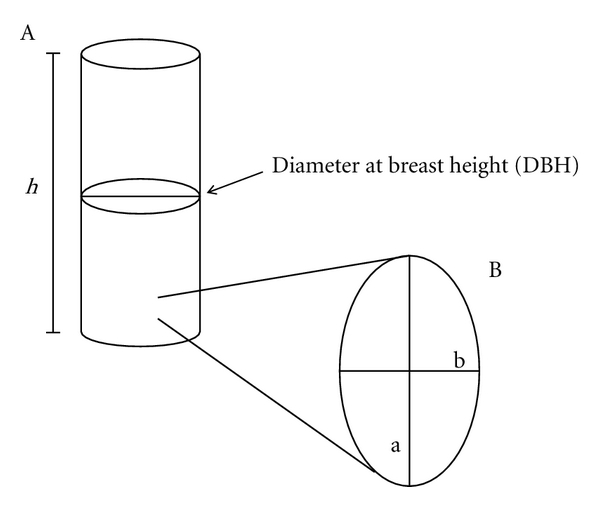
Measurements of the area of available and removed bark. (A) The cylinder represents the trunk of the plant, measured in height (*h*) and DBH. (B) The ellipse represents a scar of bark removed from the trunk, where the major axis (a) and minor axis (b) were measured.

 In the selected areas, it was not possible to find and include all 14 species selected for the study because some species did not occur or had few individuals. Therefore, the preferred species included in this stage were *Myracrodruon urundeuva, Anadenanthera colubrine*, and* Amburana cearensis*; those less-preferred were *Croton blanchetianus *and* Commiphora leptophloeos*. All individuals of these species were tagged with numbered plates and georeferenced with the inclusion criterion of being greater than 3 cm in diameter at ground level (DGL) [26]. For these species, measurements of the areas of available and removed bark were done using an adaptation of the method of Ando et al. [27]. The available bark area was calculated with the following equation: A (cm^2^) = 3.14 × DBH × *h*, as the surface area of a cylinder. Accordingly, for all tagged individuals the diameter at breast height was measured (DBH at 130 cm above ground), with a height (*h*) value of up to 2 meters, believed to be the maximum height for extraction of stem bark for medicinal use (Figure 3).

 Each instance of bark extraction (scarring) present on the stem of the individuals was considered as an extraction event, and these events were recognized in the field with the help of local experts. Once the scars were recognized, measurements of the area of extracted bark were taken by calculating the area of an ellipse (3.14 × major axis × minor axis) (Figure 3). The analysis considered all of the evidence of stem bark taken, regenerated or not, because it was not possible to discern whether the removal was recent, as each species responds differently to stem bark extraction. For example, Monteiro et al. [28] have demonstrated that the rate of regeneration can vary (months to years) in the same species, indicating the difficulty of specifying the behavior of a regenerative species without prior study.

### 2.4. Determination of Tannin Content

Of the 14 species selected for the study, the preferred species *Amburana cearensis, Anadenanthera colubrina*, *Erythrina velutina*, *Maytenus rigida*, *Mimosa tenuiflora*, *Myracrodruon urundeuva*, and* Libidibia ferrea* and the less-preferred *Schinopsis brasiliensis*, *Hymenaea courbaril*, *Handroanthus impetiginosus*, *Cereus jamacaru*, *Croton blanchetianus, *and* Spondias tuberosa *were selected for the estimation of tannins to test the second hypothesis of this research.

Of these selected species, 30 g of stem bark from three individuals of each preferred and less-preferred species were sent to the Laboratory of Natural Products of the Federal University of Pernambuco to determine the tannin content. The tannin content in the bark of each species was obtained by the radial diffusion method of Hagerman [29] adapted by Cabral et al. [30], and the experiments performed in authentic triplicates. Once in the laboratory, the bark was crushed and then macerated with methanol 50% (v/v) as a solvent. Once prepared, the mixture was administered in a solid medium in a 9-cm Petri dish, containing agarose and bovine serum albumin (BSA) in a buffer solution adjusted to pH 5.0 and consisting of 50 mM acetic acid and 60 uM ascorbic acid.

On each plate, three wells of approximately 8 *μ*L were made at a distance of 2 cm from one another and from the edges of the plates, using a punch 4 mm in diameter, where three 8 *μ*L aliquots of each plant sample were inserted. To obtain the standard curve, we used a solution of tannic acid, 25 mg/mL, of which aliquots of 2, 4, 8, 12, 16, and 20 *μ*L were inserted into wells in triplicate [30]. Subsequently, the plates were sealed with Parafilm and incubated at a temperature of 30°C in an oven for 72 hours. The halos formed in the solid medium, from the interaction of tannins with protein samples of the medium, served as indicators of tannin content. For the readings of the rings, the plates were scanned, and the program Corel Draw ×3 Version 13 was used to design two perpendicular diameters in order to obtain an average diameter for each ring [30]. The tannin concentration was then obtained from the square of the mean diameter of each ring, in *μ*g/*μ*L, from the standard curve, and, finally, the tannin content was calculated as a percentage.

### 2.5. Data Analysis

To verify the hypothesis that the area of bark collected from preferred plants is larger than that of less-preferred plants, *t*-test and the Kruskal-Wallis test were used for comparison of means, depending on data normality. The *t-*test was used to evaluate differences between the two means, a mean of the area of bark collected from the set of preferred species and another mean regarding the less-preferred species. By using the Kruskal-Wallis test, it was possible to compare the mean areas of bark collected from the species individually. In addition, we used the chi-square test to investigate differences between preferred and less-preferred plants in the proportion of individuals with evidence of extraction and without bark extraction.

The marked individuals of each species were divided into diameter classes in 3 cm intervals to record the area of bark collected and the number of individuals with evidence of extraction for each diameter class. For this purpose, subjects were grouped into classes from 1 (0–3 cm) to 27 (from 78.1 cm to 81 cm). These tests were performed in two stages: considering the individuals of each species separately and considering all marked individuals of all species.

To test the second hypothesis, according to which there is a higher content of tannin present in preferred plants compared to less-preferred plants, the plants were classified according to the amount of tannins in their bark, based on Araújo et al. [12]. According to these authors, plants with a tannin concentration greater than 10% are regarded as having high tannin content, and those with less than 10% a low tannin content. Since the radial diffusion method used in this study decreases to about half the tannin content obtained by standard methods [30], the categories used were adapted to high concentration (>5%) and low concentration (<5%). The *G* test was used to test differences between preferred and less-preferred species with regard to the proportion of plants with high and low tannin content. The species that had a tannin content not detected were considered to have low content (<5%) because null values do not mean the absence of these compounds in the bark, as the radial diffusion method has low sensitivity [30]. All tests were performed using BioEstat 5.0 [31].

## 3. Results

### 3.1. Evidence of Bark Extraction of Preferred and Less-Preferred Plants

In the three areas selected for this study, 26 individuals of the species *Myracrodruon urundeuva *were marked, of which nine individuals had evidence of bark extraction, totaling 31 extraction events; 16 individuals of *Amburana cearensis *were marked, with five individuals showing evidence of bark extraction and a total of 16 events of bark extraction. For the species *Commiphora leptophloeos*, 175 individuals were marked, with only two individuals with evidence of bark extraction for a total of two extraction events; for *Croton blanchetianus*, 99 individuals were marked, but there was no evidence of bark extraction for this species. Finally, for the species *Anadenanthera colubrina*, 121 individuals were marked, and 13 individuals presented evidence of bark extraction for a total of 25 extraction events.

Only individuals that showed evidence of extraction were included in the analysis. In this case, the species that had the largest collected bark surface were *Myracrodruon urundeuva* (aroeira) and *Amburana cearensis* (imburana-açu) with means ($\overset{̅}{x}$) and standard deviations (*σ*) of 2025.8 cm^2^  ± 2181.6 cm^2^ and 2036.4 cm^2^  ± 1931.9 cm^2^of bark collected, respectively. These were followed by *Anadenanthera colubrina* (angico) with 1497.4 cm^2^  ± 1372.8 cm^2^ of bark collected. However, *Commiphora leptophloeos* (imburana-brava) had a mean and standard deviation of 579.3 cm^2^  ± 219.8 cm^2^, with low values of collected bark area. No significant differences were found between the mean areas of bark collected between species (*H* = 2.58, *P* > 0.05).

From the marked individuals of preferred species, the total area of bark collected was 116,898.7 cm^2^, with a mean ($\overset{̅}{x}$) and standard deviation (*σ*) of 1771.2 cm^2^  ± 1636.9 cm^2^. However, for less-preferred species, only two individuals of *Commiphora leptophloeos* showed evidence of extraction, with a total area of bark collected of 1,158.6 cm^2^ and mean and standard deviation of 579.3 cm^2^  ± 219.8 cm^2^. In comparisons between these averages, there was very significant difference (*t* = 4.68, *P* < 0.01), indicating that preferred plants have a greater area of bark collected than less-preferred plants. This result supports the hypothesis of this research, showing that preferred plants listed by the informants of the Carão community suffer more use pressure.

We found a total area of bark available of 228.9 m^2^, and the species *Amburana cearensis*, *Commiphora leptophloeos* and *Myracrodruon urundeuva* showed a larger area of bark available, with means ($\overset{̅}{x}$) and standard deviation (*σ*) of 8487.5 cm^2^  ± 2651.4 cm^2^, 6659.5 cm^2^  ± 2467.8 cm^2^, and 6118.4 cm^2^  ± 2731.1 cm^2^, respectively. These species were followed by *Anadenanthera colubrina* and *Croton blanchetianus*, with means and standard deviation of 4985.9 cm^2^  ± 2878.6 cm^2^ and 2343.9 cm^2^  ± 1258.7 cm^2^, respectively. Combining the information obtained from the areas of bark removed and available, we found that species with larger area of bark available does not always have a larger area of bark collected, indicating that the collection does not appear to be related to resource availability.

**Figure 4:**
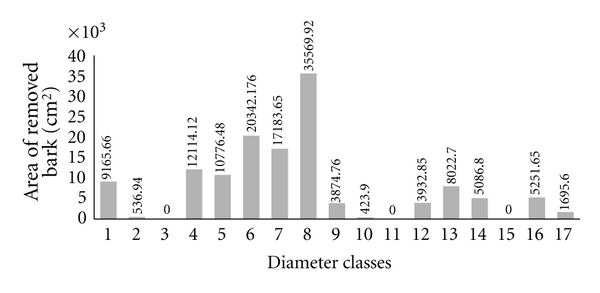
Values of the area of removed bark, divided into diameter classes, of the individuals in the community from the Carão, Altinho, northeastern Brazil. The classes, at intervals of 3 cm, correspond from 1 (0–3 cm) to 17 (48.1–51 cm). The numbers above the bars correspond to areas of bark extracted for each diameter class indicated in cm^2^.

By analyzing the extraction of the bark of the species by diameter classes, we observed that the highest values for area of bark extracted are concentrated in diameter classes 4 (9.1 cm to 12 cm), 5 (12.1 cm to 15 cm), 6 (15.1 cm to 18 cm), 7 (18.1 cm to 21 cm), and 8 (21.1 cm to 24 cm) (Figure 4). These diameters can be considered small, given that the largest individual observed was 78 cm in diameter (in the case of *Anadenanthera colubrina* individuals). However, apart from this individual, the largest individuals reached a diameter of 50 cm. In this case, the major bark extraction areas occurred in diameter classes from small to intermediate.

**Figure 5:**
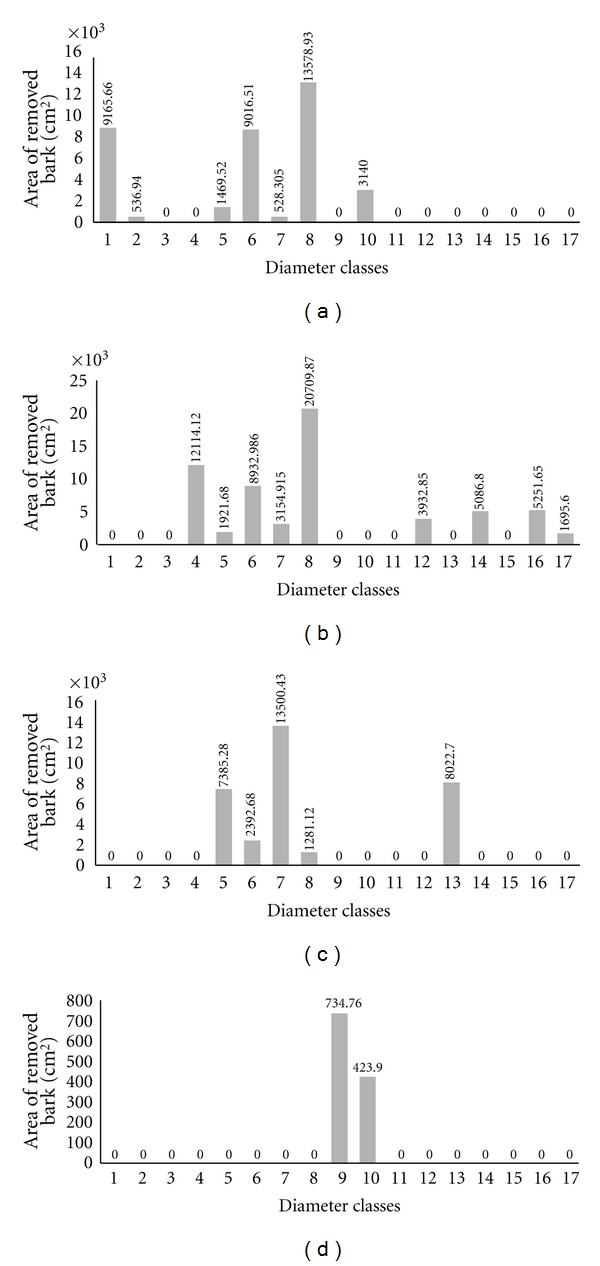
Area of bark extracted, distributed by diameter classes, of individuals of each species separately in the community of Carão, Altinho, northeastern Brazil. (a)* Anadenanthera colubrina* (Vell.) Brenan, (b) *Myracrodruon urundeuva *Allemão, (c) *Amburana cearensis* (Allemão) AC Sm, (d) *Commiphora leptophloeos* (Mart.) JB Gillett. The classes, at intervals of 3 cm, correspond from 1 (0–3 cm) to 17 (48.1–51 cm). The numbers above the bars correspond to areas of bark extracted for each diameter class indicated in cm^2^.

 Investigating each species separately, we observed that they followed the same general pattern; that is, they presented a greater area of bark extracted in individuals of small to intermediate diameters. For example, for *Anadenanthera colubrina*, individuals of classes 1, 6, and 8 presented the highest areas of bark extracted for the species (Figure 5(a)); a similar case was found for *Myracrodruon urundeuva*, with classes 4, 6, and 8 (Figure 5(b)) having the highest areas of bark extracted. The diameter classes with larger areas of bark extracted were 5, 7 and 13 for individuals of *Amburana cearensis* (Figure 5(c)), and 9 and 10 for *Commiphora leptophloeos* (Figure 5(d)).

**Figure 6:**
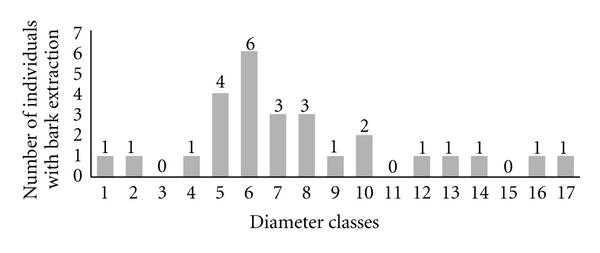
Distribution of studied individuals, with evidence of bark extraction, in diameter classes, in the community of Carão, Altinho, northeastern Brazil. The classes, at intervals of 3 cm, correspond from 1 (0–3 cm) to 17 (48.1–51 cm). The numbers above the bars correspond to areas of bark extracted for each diameter class indicated in cm^2^.

Figure 6 shows the number of individuals with evidence of extraction for each diameter class, considering all marked individuals of the species studied. There are more individuals with extracted bark in diameter classes 5, 6, 7, 8, and 10, which for most species are considered small and intermediate diameters.

 Considering only the preferred species, a total of 27 individuals presented bark extraction, as opposed to 136 individuals that had no evidence of extraction. However, in less-preferred species, only two individuals presented bark extraction, as opposed to 272 with no evidence of extraction. The results of chi-square analysis showed that the proportion of individuals with and without bark extraction depends on the preference of the species (*X* ^2^ = 41.35, *P* < 0.0001) in the sense that preferred plants have a greater number of individuals with evidence of bark extraction in relation to the less-preferred species.

### 3.2. Comparison of Tannin Content between Preferred and Less-Preferred Species

The species showing the highest levels of tannins were the preferred species, such as *Mimosa tenuiflora *and* Anadenanthera colubrina*, with 12.58% and 8.24% of tannin content, respectively, and the species *Myracrodruon urundeuva *and* Libidibia ferrea*, with 6.88%, and 6.24% respectively. The preferred species *Amburana cearensis, Erythrina velutina*, and* Maytenus rigida* did not present quantifiable values, as the method could not detect their tannin levels. In turn, the less-preferred species showed lower tannin levels, such as *Schinopsis brasiliensis* with 5.53%, *Hymenaea courbaril* with 2.35%, *Croton blanchetianus* with 2.47%, and *Spondias tuberosa* with 1.51%, unlike the species *Handroanthus impetiginosus *and* Cereus jamacaru* that did not present quantifiable values (Table 2).

By analyzing the species in which the tannin content was quantified, it is possible to observe that the preferred species had higher tannin content than the less-preferred species. However, no significant differences were found between preferred and less-preferred species in the proportion of plants with high (>5%) and low (<5%) tannin content (*G* = 2.09, *P* > 0.05). This result rejects one of the hypotheses of this research, indicating that the preference of a plant for the treatment of inflammation does not appear to be linked to its tannin content.

## 4. Discussion

### 4.1. Evidence of Bark Extraction in Preferred and Less-Preferred Plants

According to the results, preferred plants showed a larger area of bark extraction and a larger number of individuals with evidence of extraction than less-preferred plants. This pattern has been found in other ethnobotanical studies in the Caatinga, including that of Ramos et al. [32], who found a high correlation between the preference for one species as fuel and its usage frequency in timber use, indicating that preference is used as a criterion for the effective use of the resource. Other studies have found similar results, such as that of Chettri and Sharma [33], who made a list of preferred species for use as fuel according to informants from communities in the Khangchendzonga Biosphere Reserve, India. By comparing that list with the extraction results for those species obtained by Chettri et al. [34] in the same area of study, it was found that a species with the highest preference among responders also showed the highest extraction, indicating that greater use pressure may be related to the preference for a given resource. It can also be found in the literature that for a given species, the preference of a population for individuals of certain diameter classes can also lead to a greater use pressure on those classes [35]. According to these authors, who conducted a study on collection patterns for *Anadenanthera colubrina*, an absence of individuals was observed in diameter classes considered by informants as preferred for use as stakes and as firewood.

Some works have referred to a greater potential use of preferred species, but they did not investigate the extraction of individuals as proposed by this paper. Examples of such investigations include de Albuquerque and Andrade [10], who conducted an ethnobotanical study in a Caatinga community and observed that preference is restricted to a small number of species and that these, in turn, may experience concentrated use pressure. Prance et al. [36] argued that preferred plants are used more than less-preferred plants for the same use but did not conduct an investigation assessing the levels of extraction of individuals. In this respect, with the information in this research, it is confirmed that for the study area, the preference of a species leads to greater use pressure of this resource. This information raises important implications for the utilitarian redundancy model.

With the investigation of the use pressure on plant species, the utilitarian redundancy model proposes two situations involving redundant and less redundant categories [9]. The results of this research validate the status of the second situation of the model, which states that for a category highly redundant in the presence of preferred species, the use pressure will be shifted to those species, increasing the pressure of use for this utilitarian category by increasing the number of preferred species. These results do not indicate that the first situation of the model is not valid. For example, one can find situations in which a redundant utilitarian category does not present species preference by the informants. In this case, a greater number of species in that category may lead to a mutual reinforcement and thus a lower pressure of use among individual species [9].

The preferred species *Myracrodruon urundeuva *and* Amburana cearensis*, followed by *Anadenanthera colubrina,* presented the largest areas of bark extraction. These preferred species have been reported in several studies as being important for local communities in various Caatinga environments [9, 37, 38]. de Albuquerque et al. [8] and Monteiro et al. [35] showed that the species *Myracrodruon urundeuva *and* Anadenanthera colubrina* have a high versatility among native plants, demonstrating that these plants typically have multiple uses [39] and are extensively used for various therapeutic treatments [6]. Although de Albuquerque et al. [8] claimed that a high versatility did not necessarily indicate a greater harvest pressure, a potential use pressure on these species can be expected.

The classes of small and intermediate diameter had the largest areas of bark extracted and the largest number of individuals with evidence of extraction. Monteiro et al. [35] found similar results when observing a decrease in individuals of the intermediate class of *Anadenanthera colubrina*, which are considered as favorites for use as stakes and fuel. Similarly, Lins Neto et al. [40] found a higher percentage of bark extraction in *Myracrodruon urundeuva* individuals with small diameters when investigating the use of this species by residents of two local populations of the Caatinga. This discussion should be seen in perspective, as the studies discussed above were conducted with uses other than medicinal. To decrease the use pressure concentrated in these classes, it is suggested that the extraction of bark is directed to individuals of greater size, which can better withstand the extraction, as there do not seem any differences in the therapeutic efficiency of bark, as measured by tannin content, when collected from individuals of different diameter classes [30, 41]. These authors found no differences in the amount of tannins among diameter classes for the species of *Myracrodruon urundeuva *and* Syderoxilon obtusifolium*.

### 4.2. Comparison of Tannin Content between Preferred and Less-Preferred Species

Tannins are phenolic compounds, products of secondary metabolism in plants, which protect them from external agents, such as attacks from herbivores [42]. According to Monteiro et al. [43], so far there are only a few studies investigating the activity of these compounds for the treatment of diseases from medicinal plants. For the treatment of inflammation, tannins form complexes with proteins and polysaccharides [44] which may, for example, form protective layers on injured epithelial tissues [45] that present antimicrobial and antifungal activity [46]. Therefore, tannins may exert anti-inflammatory activity in epithelial tissues.

When investigating the tannin content in the preferred species, it was observed that although the species *Myracrodruon urundeuva *and* Libidibia ferrea* have the highest tannin concentrations, along with *Mimosa tenuiflora *and* Anadenanthera colubrina*, there are still few studies on the biological activity of these species *in vitro *or* in vivo* for the treatment of inflammation. For example, in the few studies that investigate the anti-inflammatory properties of *Myracrodruon urundeuva*, anti-inflammatory activity was observed in the treatment of periodontitis [47], colitis [48], ulcers [49], and inflammation of the genital tract [50] in animal models.

It has been shown that the anti-inflammatory activity of *Myracrodruon urundeuva *and* Anadenanthera colubrina* is attributed to the large amount of tannins present in their bark [7, 12]. These species are also important because they are widely used by various local communities of the Caatinga for the treatment of inflammation [7, 10, 35]. Considering the importance of these species and the few pharmacological studies associated with them, it is important for future pharmacological studies to investigate the anti-inflammatory properties of these plants because they may indicate new medicinal potential.

According to the results obtained, it was expected that the tannin content present in preferred species would be significantly higher than that in less-preferred species because the tannin compound may account for the anti-inflammatory activity, but this was not the case. These results do not negate the importance of tannins in the selection of plants by populations of the Caatinga for the treatment of inflammation [7, 12], but indicate that the preference of a plant as anti-inflammatory does not seem to be linked to its total tannin content. It is also possible that other phenolic compounds are involved in the anti-inflammatory activity of these plants.

## 5. Conclusions

Few studies have investigated the relationship between preference and use, and these are focused on the timber and fuel use, for example. For medicinal use, properly anti-inflammatory use, this research showed that preferred plants actually had a higher pressure than less-preferred plants. From these results, the second situation of the utilitarian redundancy model is sustained in the sense that the preference for plants increases the use pressure in redundant utilitarian categories. However, this conclusion is limited to the community studied and the anti-inflammatory category. Other studies with a similar approach should be conducted in other regions and with different medical categories in order for more robust conclusions to be reached.

The results of this research, although focused on the anti-inflammatory category, showed that the species *Myracrodruon urundeuva*, *Amburana cearensis*, and* Anadenanthera colubrina* experienced higher rates of extraction. This fact, together with information from other studies conducted in local populations integrated in the Caatinga environment, shows that these species should be targeted in future management programs.

According to the data obtained, we cannot affirm that tannin content is a criterion for indicating preference, as there were no differences in tannin content between preferred and less-preferred species. However, future studies should be conducted because the present research examined only the total tannin content in the bark of the species studied, and differences can be obtained whether the content between tannins classes was studied.

## Figures and Tables

**Table 1:** Plants suitable for the treatment of inflammation based on the preferences of the informants in the rural community of Carão, Altinho, northeastern Brazil. In parenthesis, the synonym by which the species is more widely known.

Family	Species	Popular name	Voucher
*Preferred*			
Anacardiaceae	*Myracrodruon urundeuva* Allemão	Aroeira	50872
Caesalpiniaceae	*Libidibia ferrea* (Mart. ex Tul.) L. P. Queiroz (*Caesalpinia ferrea *Mart.)	Jucá	48664
Celastraceae	*Maytenus rigida* Mart.	Bom-nome	46182
Fabaceae	*Amburana cearensis* (Allemão) A. C. Sm.	Imburana-açu	50486
	*Erythrina velutina* Willd.	Mulungu	46180
Mimosaceae	*Anadenanthera colubrina* (Vell.) Brenan	Angico	48663
	*Mimosa tenuiflora* (Willd) Poir.	Jurema-preta	50871

*Less preferred*			
Anacardiaceae	*Schinopsis brasiliensis* Engl.	Baraúna	49640
	*Spondias tuberosa* Arruda	Umbu	48652
Bignoniaceae	*Handroanthus impetiginosus *(Mart. ex DC.) Mattos (*Tabebuia impetiginosa *(Mart. ex DC.) Standl.)	Pau-d'arco-roxo	50481
Burseraceae	*Commiphora leptophloeos* (Mart.) J. B. Gillett	Imburana-brava	48657
Cactaceae	*Cereus jamacaru* DC.	Mandacaru	*nc*
Caesalpiniaceae	*Hymenaea courbaril *L.	Jatobá	*nc*
Euphorbiaceae	*Croton blanchetianus* Baill.	Marmeleiro	48653

*nc*: not collected.

**Table 2:** Tannin content, expressed as a percentage, of the preferred and less-preferred species studied in the community of Carão, Altinho, northeastern Brazil.

Species	Tannin content (%)
Preferred	
*Amburana cearensis* (Allemão) A. C. Sm.	*nd*
*Anadenanthera colubrina* (Vell.) Brenan	8.24
*Erythrina velutina* Willd.	*nd*
*Maytenus rigida* Mart.	*nd*
*Mimosa tenuiflora* (Willd) Poir.	12.58
*Myracrodruon urundeuva* Allemão	6.88
*Libidibia ferrea* (Mart. ex Tul.) L. P. Queiroz	6.24
Less preferred	
*Schinopsis brasiliensis* Engl.	5.53
*Hymenaea courbaril *L.	2.35
*Handroanthus impetiginosus *(Mart. ex DC.) Mattos	*nd*
*Cereus jamacaru* DC.	*nd*
*Croton blanchetianus* Baill.	2.47
*Spondias tuberosa* Arruda	1.51

*nd*: not detected.
